# Multi-omics analyses reveal interactions between the skin microbiota and skin metabolites in atopic dermatitis

**DOI:** 10.3389/fmicb.2024.1349674

**Published:** 2024-03-15

**Authors:** Kaikai Huang, Fang Li, Yingyao Liu, Baoying Liang, Pinghua Qu, Linlin Yang, Shanshan Han, Wenjun Li, Xiumei Mo, Lei Dong, Ying Lin

**Affiliations:** ^1^Department of Dermatology, The Second Affiliated Hospital of Guangzhou University of Chinese Medicine, Guangzhou, China; ^2^The Second Clinical Medical College, Guangzhou University of Chinese Medicine, Guangzhou, China; ^3^Department of Clinical Laboratory, The Second Affiliated Hospital of Guangzhou University of Chinese Medicine, Guangzhou, China; ^4^School of Life Sciences, Sun Yat-sen University, Guangzhou, China; ^5^State Key Laboratory of Dampness Syndrome of Chinese Medicine, The Second Affiliated Hospital of Guangzhou University of Chinese Medicine, Guangzhou, China; ^6^Guangdong Provincial Clinical Research Center for Chinese Medicine Dermatology, Guangzhou, China; ^7^Guangdong Provincial Key Laboratory of Chinese Medicine for Prevention and Treatment of Refractory Chronic Diseases, Guangzhou, China

**Keywords:** atopic dermatitis, skin microbiome, skin metabolome, correlation analysis, purine metabolism, phenylalanine metabolism

## Abstract

**Introduction:**

Atopic dermatitis (AD) is one of the most common inflammatory skin diseases. Skin microecological imbalance is an important factor in the pathogenesis of AD, but the underlying mechanism of its interaction with humans remains unclear.

**Methods:**

16S rRNA gene sequencing was conducted to reveal the skin microbiota dynamics. Changes in skin metabolites were tracked by LC–MS metabolomics. We then explored the potential mechanism of interaction by analyzing the correlation between skin bacterial communities and metabolites in corresponding skin-associated samples.

**Results:**

Samples from 18 AD patients and 18 healthy volunteers (HVs) were subjected to 16S rRNA gene sequencing and LC–MS metabolomics. AD patients had dysbiosis of the skin bacterial community with decreased species richness and evenness. The relative abundance of the genus *Staphylococcus* increased significantly in AD, while the abundances of the genera *Propionibacterium* and *Brevundimonas* decreased significantly. The relative abundance of the genera *Staphylococcus* in healthy females was significantly higher than those in healthy males, while it showed no difference in AD patients with or without lesions. The effects of AD status, sex and the presence or absence of rashes on the number of differentially abundant metabolites *per capita* were successively reduced. Multiple metabolites involved in purine metabolism and phenylalanine metabolism pathways (such as xanthosine/xanthine and L-phenylalanine/trans-cinnamate) were increased in AD patients. These trends were much more obvious between female AD patients and female HVs. Spearman correlation analysis revealed that the genus *Staphylococcus* was positively correlated with various compounds involved in phenylalanine metabolism and purine metabolic pathways. The genera *Brevundimonas* and *Lactobacillus* were negatively correlated with various compounds involved in purine metabolism, phenylalanine metabolism and sphingolipid signaling pathways.

**Discussion:**

We suggest that purine metabolism and phenylalanine metabolism pathway disorders may play a certain role in the pathogenic mechanism of *Staphylococcus aureus* in AD. We also found that females are more likely to be colonized by the genus *Staphylococcus* than males. Differentially abundant metabolites involved in purine metabolism and phenylalanine metabolism pathways were more obvious in female. However, we should notice that the metabolites we detected do not necessarily derived from microbes, they may also origin from the host.

## 1 Introduction

Atopic dermatitis (AD) is one of the most common inflammatory skin diseases and clinically manifests as an eczema-like rash with pruritus (Ständer, 2021; Zhang et al., 2023). The prevalence of AD is 15–20% among children and up to 10% among adults, making AD the 15th most common nonfatal disease and the skin disease with the highest disease burden in disability-adjusted life years (Ständer, 2021; Schuler et al., 2023). In addition, AD may be associated with an increasing prevalence of psychosocial disorders, such as depression, anxiety, sleep disorders and suicidal ideation (Kage et al., 2020). Epidermal barrier dysfunction, skin microecological imbalance and immune disorders, which are dominated by type 2 inflammation, contribute to the outbreak of AD (Langan et al., 2020).

The skin microbiota has a complex interaction with the host (Chen et al., 2018). In recent years, with the application of high-throughput DNA sequencing technologies based on two principal methods (16S rRNA gene sequencing and metagenomics sequencing), the study of the AD skin microbiome has shown great progress (Byrd et al., 2018; Ghosh et al., 2018). Skin microbiota disturbances characterized by increased *Staphylococcus aureus* colonization and decreased microbiota diversity occurred in both lesion and nonlesion sites of AD patients (Koh et al., 2022). *S. aureus* may destroy the skin barrier and induce an inflammatory response (Langan et al., 2020). The local Th2 immune response further diminishes barrier function and facilitates the growth of *Staphylococcus,* especially *S. aureus* (Langan et al., 2020; Schuler et al., 2023). However, the composition of the skin microbiome is complex, and the specific mechanism of its interaction with the host remains unclear.

Metabolomics is a technique that reflects the phenotypic outcome of biological activities and elucidates the relationship between metabolite changes and physiological/pathological changes (Zhang et al., 2023). Skin cells and glandular secretions, skin resident microbiota, and external environmental factors (such as cosmetics or pollution) can affect the composition of skin metabolites (Afghani et al., 2022). The metabolomic profile of 15 AD patients and 17 controls was studied through skin punch biopsies, which revealed that a total of 77 metabolites differed significantly between the lesional skin of atopic dermatitis, nonlesional skin of atopic dermatitis and skin of controls (Ilves et al., 2021). Until now, much of the metabolomics research on AD skin has focused on the proportion of changes in the lipid composition of the skin due to its role in the structural integrity of the skin barrier (Emmert et al., 2021; Zhang et al., 2023).

Theoretically, the skin microbiome and the skin would constantly interact with surrounding skin metabolites. The identification of AD skin metabolites may help to determine the changes in host status and the influence of the skin microbiome on the local skin. Several previous studies have explored the interactions between the skin microbiota and skin metabolites in AD. AD skin has an increase in free fatty acids (FFA), and this effect appears to be *S. aureus*-dependent (Emmert et al., 2021). Saturated shorter-chain FFA are negatively correlated with *Staphylococci* (Emmert et al., 2021), and long-chain FFA are decreased in *S. aureus*-colonized AD skin (Li et al., 2017). Shorter fatty acids can more easily traverse the skin to acidify it, and *S. aureus* does not grow well in acidic healthy skin pH conditions (Afghani et al., 2022). However, lipids are not the only metabolites present in the skin, and an increasing number of studies are starting to turn from lipids to other molecules in AD (Afghani et al., 2022). Herein, we performed 16S rRNA gene sequencing of the skin microbiome and untargeted metabolomics analysis of AD patients’ skin samples, and searched for metabolites closely related to the skin microbiome of AD patients through association analysis. We also explored the effects of sex and rash status on the results through subgroup analysis. We established a correlation map of the skin microbiome and skin metabolome in AD patients and healthy people. These results highlight the role of the skin microbiome in regulating overall metabolism and provide new insights into the pathological mechanisms of AD.

## 2 Materials and methods

### 2.1 Study population

AD patients and healthy volunteers (HVs) were recruited from Guangdong Provincial Hospital of Traditional Chinese Medicine from October 2019 to May 2021. The inclusion criteria were as follows: over 18 years of age; patients meeting the Williams AD diagnostic criteria (Williams et al., 1994), with or without rash in both elbow fossa; and volunteers with no skin disease. The exclusion criteria were as follows: patients who received systemic immunosuppressive agents, biological agents, antibiotics, antifungals and glucocorticoid drugs, or ultraviolet rays and other systematic treatment in the previous 1 month; participants with severe kidney or liver damage, mental illness, or other serious organ disease; and participants who had used topical glucocorticoid, anti-biological ointment, skin care product or lotion on the elbow fossa in the previous week. All clinical information and samples were obtained with informed consent. This study was conducted with the approval of the institutional review board of the Guangdong Provincial Hospital of Traditional Chinese Medicine and in accordance with the Declaration of Helsinki (World Medical Association, 2013).

### 2.2 Sample collection

**Figure 1:**
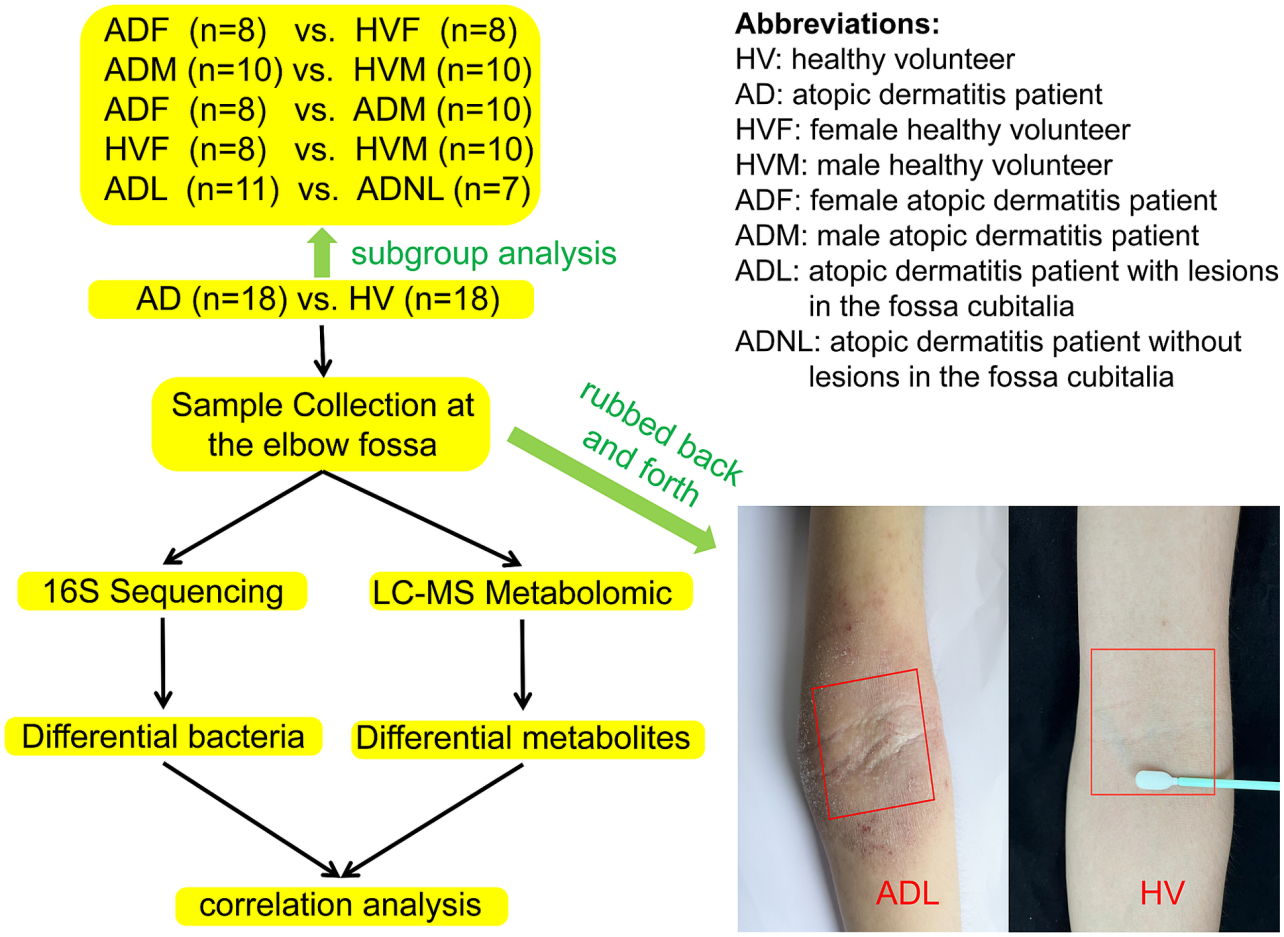
Analysis workflow.

A sterile double-sided polyester swab was soaked with 0.9% NaCl and forcefully rubbed back and forth 50 times at the elbow fossa within a range of approximately 4 cm × 4 cm (regardless of whether there were rashes at the elbow fossa). The head of each swab was then placed in a sterile liquid-holding tube, fully oscillated, and cut aseptically from the handle before closing the tube cap. The same procedure was used to sample the skin of the elbow fossa again, and the samples were stored in another sterile storage tube. The two samples were refrigerated at −80°C within 2 h until 16S rRNA high-throughput sequencing and metabolomics were performed. The workflow of this study is shown in Figure 1.

### 2.3 Microbiome DNA extraction and 16S sequencing

The DNA of the microbial community was extracted by a MagPure Stool DNA KF kit B (Magen, China) following the manufacturer’s instructions. The quality of all extracted DNA was assessed with a Qubit® dsDNA BR Assay kit (Invitrogen, United States) and agarose gel electrophoresis. Variable region V4 of the bacterial 16S rRNA gene was amplified with the common PCR primers 515F (5′-GTGCCAGCMGCCGCGGTAA-3′) and 806R (5′- GGACTACHVGGGTWTCTAAT-3′). Both forward and reverse primers were tagged with Illumina adapter, pad, and linker sequences.

The PCR products were purified using Agencourt AMPure XP beads. The validated libraries were used for sequencing on the Illumina HiSeq 2,500 platform (BGI, Shenzhen, China) following Illumina’s standard pipelines and generating 2 × 250 bp paired-end reads.

### 2.4 16S amplicon sequencing data analysis

After Illumina sequencing, barcode and primer sequences were removed. Specific tags were generated by FLASH software (version 1.2.11) according to the overlap information of the reads (Magoč and Salzberg, 2011). Tags were clustered into operational taxonomic units (OTUs) with a 97% threshold by USEARCH (v7.0.1090), where the unique OTU representative sequences can be obtained (Edgar, 2010). Chimeras were filtered by UCHIME (v4.2.40) (Edgar et al., 2011). OTU representative sequences were aligned against the database for taxonomic annotation by RDP classifier (v2.2) software (sequence identity was set to 0.6) (Wang et al., 2007). Alpha diversity and beta diversity analyses were conducted by mothur (v1.31.2) and QIIME (v1.80), respectively (Schloss et al., 2009; Caporaso et al., 2010).

### 2.5 Liquid chromatography–mass spectrometry metabolomic data collection

Samples (100 μL) were placed into an EP tube and extracted with 400 μL extract solution (methanol:acetonitrile:water 2:2:1), vortexed for 1 min, sonicated for 10 min, and incubated for 1 h at −20°C. Samples were centrifuged for 15 min at 25000 rpm at 4°C, and the supernatant was then transferred for vacuum freeze drying. The metabolites were resuspended in 200 μL of 10% methanol and sonicated for 10 min at 4°C. After centrifuging for 15 min at 25000 rpm, the supernatants were transferred to a new glass vial for further analysis. The QC samples were then mixed from each sample.

The samples were analyzed on a Waters 2D UPLC (Waters, United States) coupled to a Q Exactive mass spectrometer (Thermo Fisher Scientific, United States) with a heated electrospray ionization (HESI) source and controlled by the Xcalibur 2.3 software program (Thermo Fisher Scientific, Waltham, MA, United States), according to previously reported methods with minor modifications (Chen et al., 2021). Briefly, the mobile phase consisted of 0.1% formic acid (A) and acetonitrile (B) in positive mode and 10 mM ammonium formate (A) and acetonitrile (B) in negative mode. The column temperature was maintained at 45°C. The flow rate was 0.35 mL/min, and the injection volume was 5 μL. The mass spectrometric settings for positive/negative ionization modes were as follows: spray voltage, 3.8/−3.2 kV; sheath gas flow rate, 40 arbitrary units (arb); aux gas flow rate, 10 arb; aux gas heater temperature, 350°C; capillary temperature, 320°C.

### 2.6 LC–MS metabolomic data analyses

LC–MS/MS data processing was performed using Compound Discoverer 3.1 (Thermo Fisher Scientific, United States) software, including peak extraction, peak alignment, and compound identification. Data preprocessing, statistical analysis, metabolite classification annotations and functional annotations were performed using the metabolomics R package metaX (BGI, Shenzhen, China) and the metabolome bioinformatic analysis pipeline (Wen et al., 2017). The multivariate raw data are dimensionally reduced by principal component analysis (PCA) to analyze the groupings, trends (intra- and intergroup similarities and differences) and outliers of the observed variables in the data set (whether there is an abnormal sample). Partial least squares method-discriminant analysis (PLS-DA), the variable importance in projection (VIP) values of the first two principal components of the model, combined with the variability analysis, the fold change and Student’s *t* test were used to screen for differentially abundant metabolites.

### 2.7 Statistical analysis

Statistical analysis was performed in the R platform (v3.5.1). The differences in alpha diversity indexes were determined by Student’s *t* test. The beta diversity difference between the two groups was analyzed by analysis of similarity (ANOSIM). Differences in the relative abundance of genera between the two groups were evaluated with the Wilcoxon rank sum test. False-discovery rate (FDR) values were estimated using the Benjamini-Hochberg method to control for multiple testing. Linear discriminant analysis coupled with effect size (LEfSe) was applied to identify microorganisms that can be used to discriminate AD patients from HVs (Segata et al., 2011). A *p* value threshold cutoff at 0.05 was considered. Spearman correlation was carried out to determine the relationship between the skin microbiota and metabolites.

## 3 Results

### 3.1 Study population characteristics

After quality control, samples from 18 AD patients and 18 HVs were successfully subjected to 16S rRNA gene sequencing and LC–MS metabolomic analyses. Subgroup analysis was conducted according to whether there were rashes in the sampling site of the elbow fossa and by sex. The characteristics of the participants in each group are shown in Figure 1 and Supplementary Table S1.

### 3.2 Altered skin microbiota composition in AD patients

**Figure 2:**
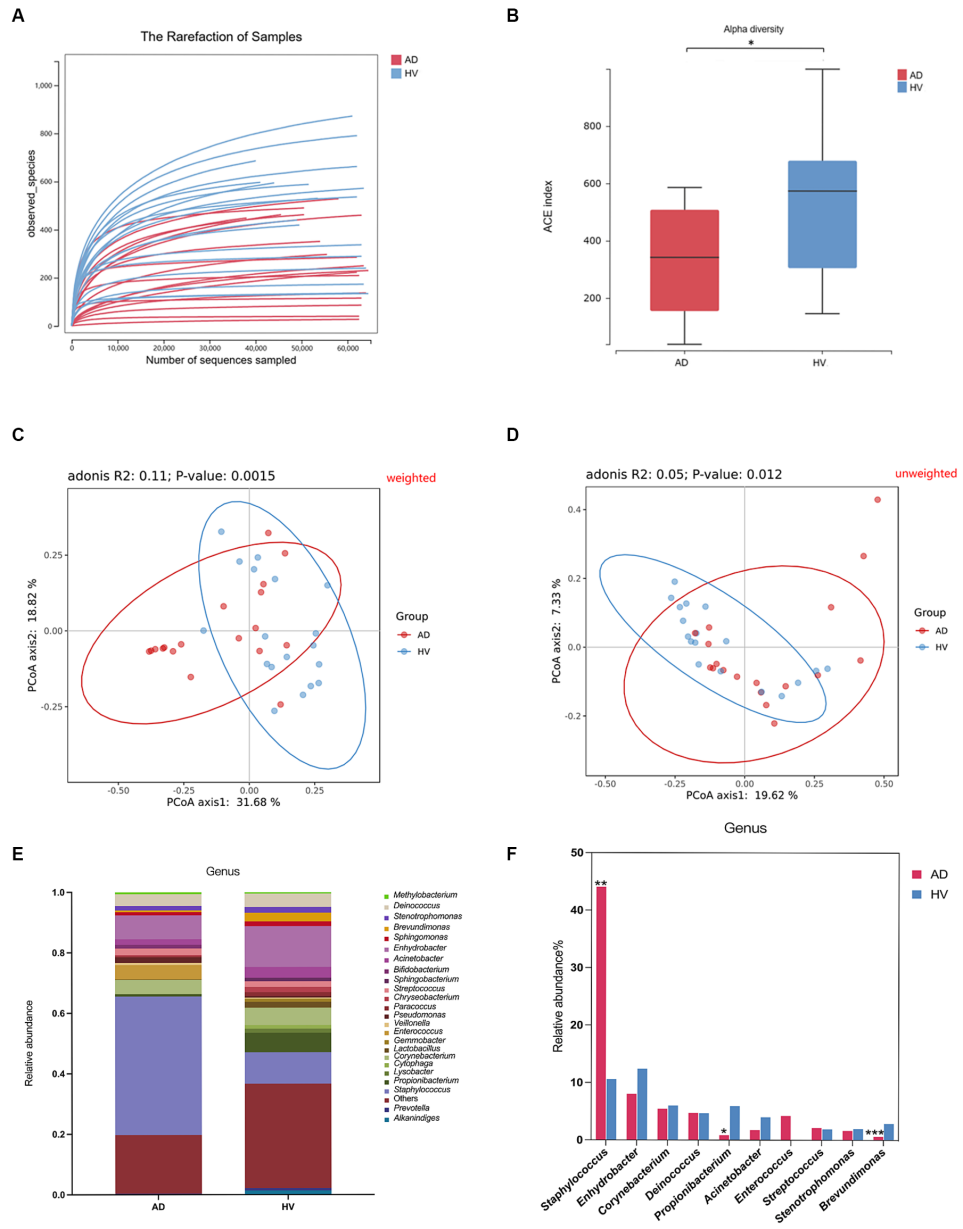
Skin microbiome diversity and structure comparison between the AD group and HV group. **(A)** Rarefaction curve of all samples. **(B)** Alpha diversity analyzed by the ACE index. **(C)** Weighted Unifrac PCoA plot. **(D)** Unweighted Unifrac PCoA plot. **(E)** Comparison of bacteria at the genus level. **(F)** Top 10 genera in relative abundance. *p < 0.05; **p < 0.01; ***p < 0.001.

In the present study, a total of 2,073,232 effective 16S rRNA gene sequencing reads were obtained from the skin samples of 18 AD patients and 18 HV, with an average of 57589.8 reads per sample (ranging from 38,199 to 64,529). A total of 3,097 OTUs were obtained according to 97% similarity. After taxonomic assignment against the Greengenes (v201305) database, they were annotated at different phylogenetic levels (Supplementary Table S2). According to the rarefaction curve (Figure 2A), the current sequencing depth and samples were sufficient for taxa identification.

Alpha diversity represents the species richness and evenness within the microbiota, while beta diversity can reflect the shared diversity within the microbiota at different ecological distances (Yang et al., 2022). The alpha diversity indexes, including the ACE index, Shannon index and Chao index, of the skin of AD patients were less than those of the HV group (*p* < 0.05) (Figure 2B; Supplementary Figures S1A,B), indicating decreased richness and evenness of the skin microbiome in AD patients. In addition, the ACE index and Chao index of female atopic dermatitis patients (ADFs) were less than those of female healthy volunteers (HVFs) (*p* < 0.05) (Supplementary Figures S1C,D). The score plot of principal coordinate analysis (PCoA) based on weighted and unweighted UniFrac distances showed differences in the composition and structure of the bacterial community between the AD group and HV group (*p* < 0.05) (Figures 2C,D). There were significant differences in beta diversity analyzed by weighted UniFrac PCoA of all subgroups (Supplementary Figures S2A–E).

There was considerable variation in the relative abundance of 569 differentially abundant bacteria at the genus level (Figure 2E). Compared with HVs, AD patients showed a significantly greater relative abundance of the genus *Staphylococcus* (44.1% vs. 10.6%) and lower levels of the genera *Propionibacterium* (0.9% vs. 5.9%) and *Brevundimonas* (0.6% vs. 2.9%) (*p* < 0.05) (Figure 2F). The relative abundances of the genera *Staphylococcus, Acinetobacter, Lactobacillus* and *Streptococcus* in HVFs were significantly higher than those in male healthy volunteers (HVMs) (*p* < 0.05) (Supplementary Figure S4A). Microbiome compositions at the genus level in other subgroups are shown in Supplementary Figures S3A–E, 4B–E.

**Figure 3:**
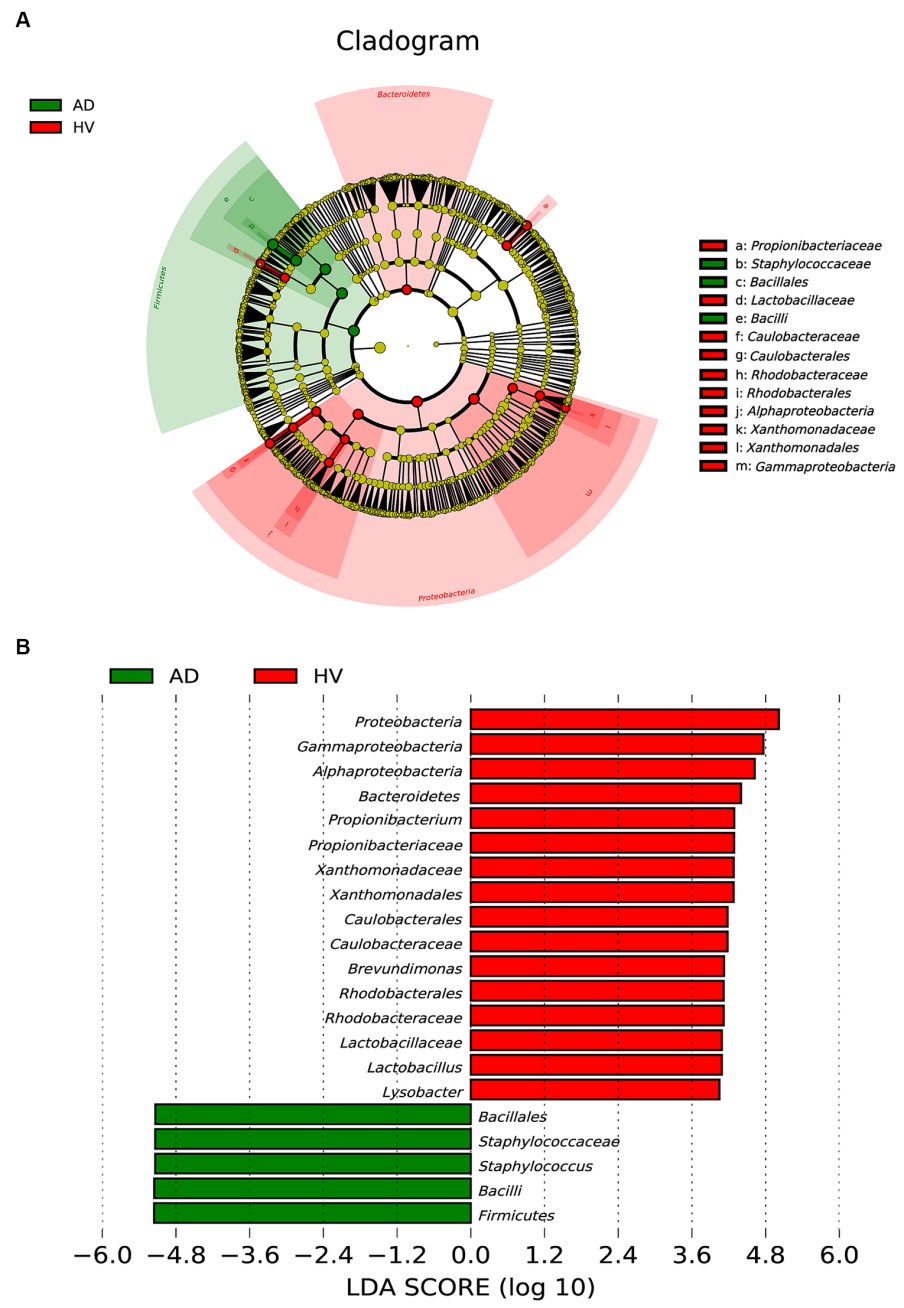
Linear discriminant analysis (LDA) effect size of the AD group and HV group. **(A)** Cladogram of LEfSe of the skin microbiome from 16S rRNA gene sequencing results. **(B)** Histogram of the LDA scores for differentially abundant microbes in AD patients and healthy controls (LDA > 4.0).

To further examine the alterations associated with AD, we conducted LEfSe analysis. The main differences were the increase in the abundance of *Bacillales* (class *Bacilli* and order *Bacillales*) in AD patients and the reduction in *Caulobacterales* (class *Alphaproteobacteria* and order *Caulobacterales*), *Rhodobacterales* (class *Alphaproteobacteria* and order *Rhodobacterales*) and *Xanthomonadales* (class *Gammaproteobacteria* and order *Xanthomonadales*) (Figure 3A). Some differences were also observed at a lower taxonomic level. AD patients showed an increase in *Staphylococcaceae* (genus *Staphylococcus*). In contrast, AD patients exhibited a loss of *Propionibacterium*, *Brevundimonas*, *Lactobacillus* and *Lysobacter* at the genus level (Figure 3B). Taken together, these data indicate alterations in the commensal skin microbiome composition in AD patients, suggesting dysregulation of the microbial community.

### 3.3 Skin metabolic profiling of AD patients

According to the information available for matching (including MS1 molecular weight, MS2 fragment spectra, column retention time and whether there are reference standards), the credibility levels of the identified substances are annotated to divide them into different credibility levels (Supplementary Table S3). In general, a total of 23,700 features and 3,867 metabolites (level 1–5) were identified (Supplementary Table S4). All metabolites were classified into several classes, including compounds with biological roles (benzene and derivatives, amino acids, peptides, organic acids, carbohydrates, etc.), lipids (polyketides, fatty acyls, etc.), phytochemical compounds (terpenoids, alkaloids, flavonoids, etc.) and others. KEGG pathway analysis was conducted and showed that metabolism (including amino acid metabolism and lipid metabolism) was the main function of the metabolites.

**Figure 4:**
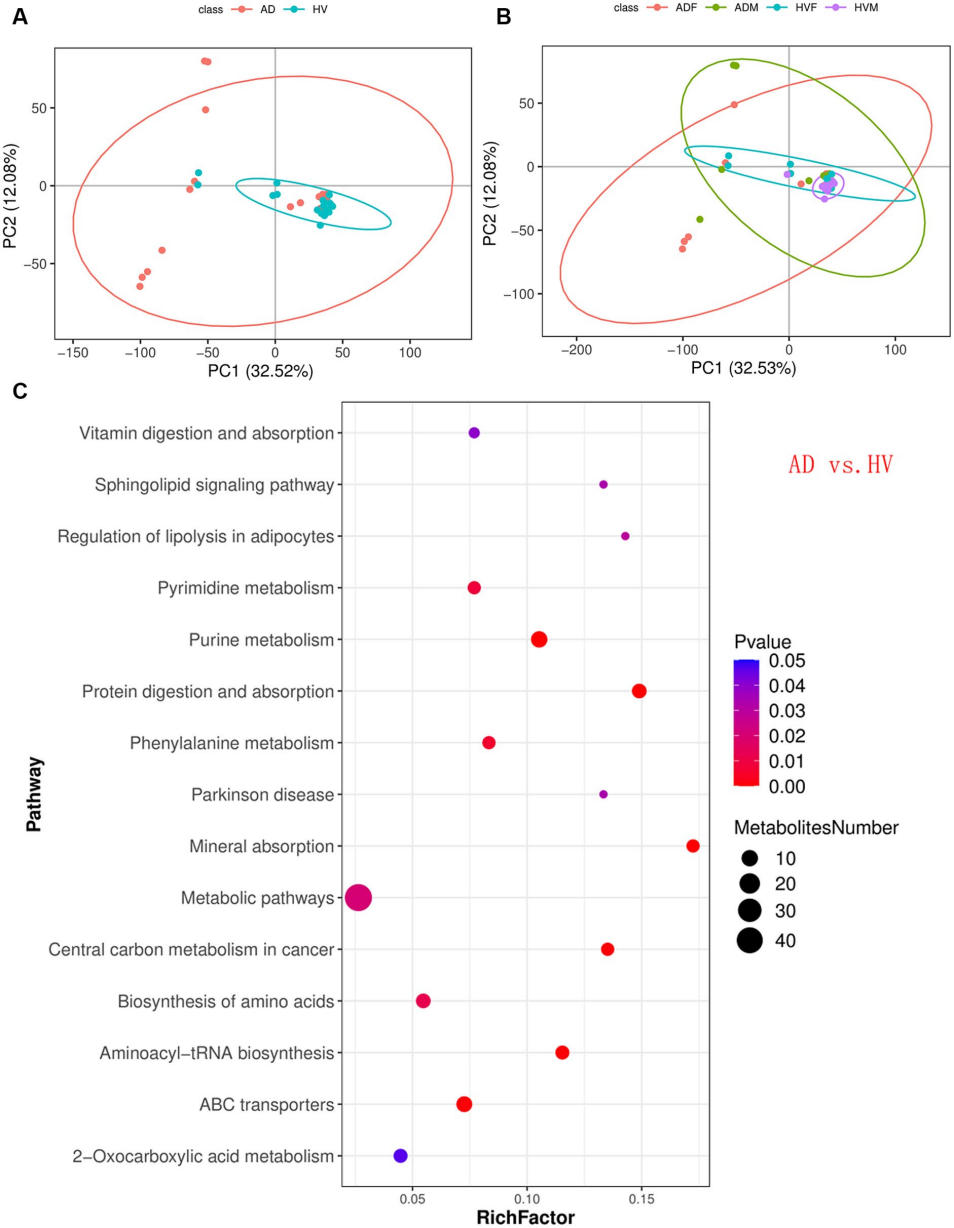
Skin metabolomic profiling. **(A)** PCA revealed that the distribution of samples in the HV group was more concentrated than that in the AD group. **(B)** PCA revealed that the distributions of samples in the ADM group and HVM group were more concentrated than those in the ADF group and HVF group, respectively. **(C)** Bubble plot of the metabolic pathway enrichment analysis results of the AD group and HV group.

PCA, a multivariate technique, was used to determine whether samples from different groups could be segregated based on their metabolic profiles. The PCA results showed that the distribution of samples in the HV group was more concentrated than that in the AD group (Figure 4A). Similarly, the distributions of samples in the male atopic dermatitis patient (ADM) group and HVM group were more concentrated than those in the ADF group and HVF group, respectively (Figure 4B).

A total of 773 differentially abundant metabolites (VIP ≥ 1; fold-change ≥1.2 or ≤ 0.83; *p* < 0.05) between the AD group and HV group, at the credibility level of level 1 to level 4, were obtained (Supplementary Table S5). Similarly, differentially abundant metabolites in other subgroups were calculated, and the number of differentially abundant metabolites in each subgroup is shown in Table 1. Compared with the total number, the *per capita* number was more appropriate to reflect the effect of different influencing factors. We found that the number of differentially abundant metabolites *per capita* could be divided into three levels. Under the same gender, the number of differentially abundant metabolites *per capita* between AD patients and HVs was the largest, at more than 20. In the same state of illness or health, the number of differentially abundant metabolites *per capita* between different genders was in the middle, approximately 10. The number of differentially abundant metabolites *per capita* in AD patients with or without rashes was the lowest, approximately 5. Thus, we inferred that the state of AD is the most important factor that causes significant changes in skin metabolites. The gender factor ranked second. The presence or absence of rashes in AD has relatively little influence on skin metabolites.

**Table 1:** The number of differentially abundant metabolites in each group.

Group	Up	Down	Total number	*Per capita* number
AD vs. HV	334	439	773	21.5
ADF vs. HVF	213	161	374	23.4
ADM vs. HVM	189	300	489	24.5
ADF vs. ADM	93	65	158	8.8
HVF vs. HVM	66	128	194	10.8
ADL vs. ADNL	49	32	81	4.5

Differentially abundant metabolite screening conditions: (1) VIP of the first two principal components of the PLS-DA model ≥ 1; (2) fold-change ≥ 1.2 or ≤ 0.83; (3) *p* < 0.05.

**Figure 5:**
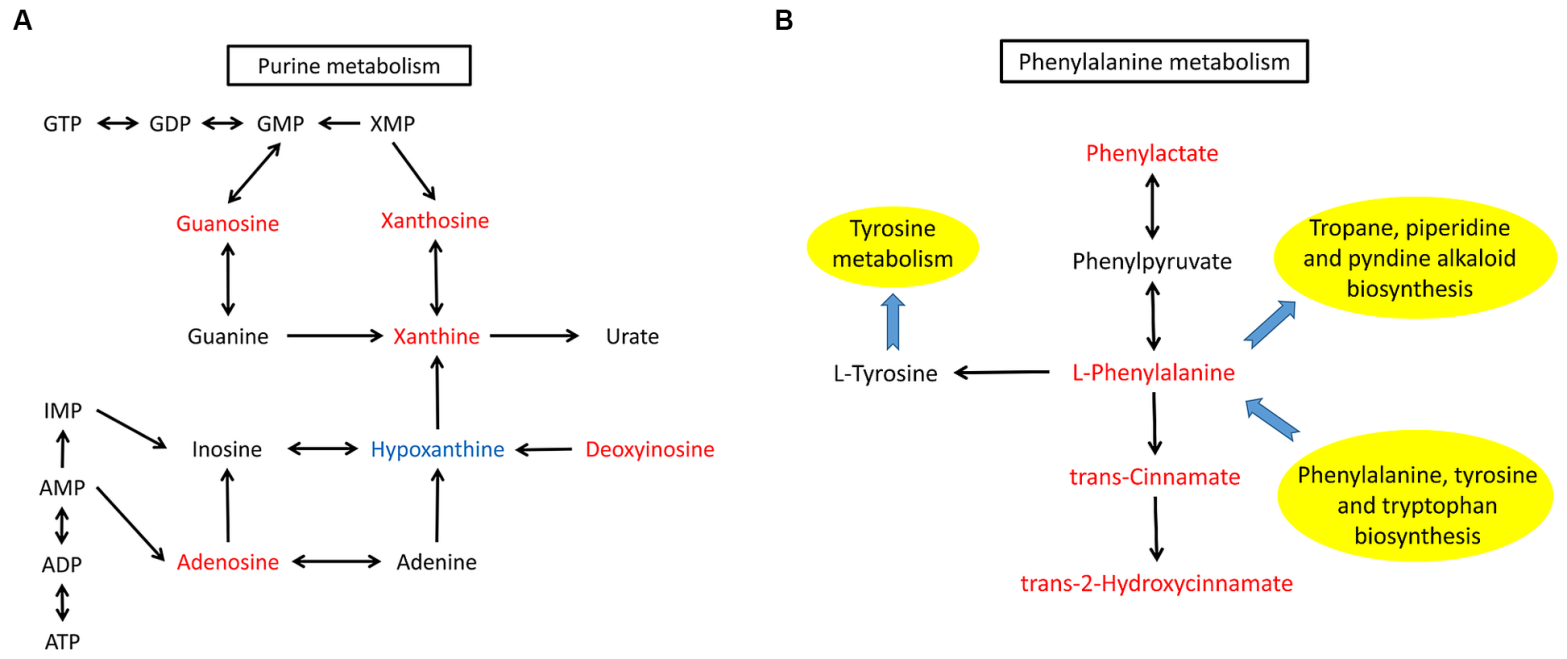
Altered metabolites and metabolic pathways observed in AD patients. **(A)** The purine metabolism pathway. **(B)** The phenylalanine metabolism pathway. Differentially abundant metabolites are shaded in red or blue. Red represents elevation compared with the HV group, and blue indicates a decrease compared with the HV group. The black arrow represents molecular interaction or relation. The blue arrow indicates the link to/from another KEGG pathway map.

KEGG pathway analysis of the differentially abundant metabolites between the AD group and HV group was conducted to uncover the metabolic pathway alterations (Figure 4C). The dot size of metabolic pathways was the largest, indicating the largest amounts of differentially abundant metabolites annotated to metabolic pathways. Furthermore, the differentially abundant metabolites of each enriched pathway were analyzed, and it was found that there were upstream and downstream cascades in the purine metabolism and phenylalanine metabolism pathways (Figures 5A,B). The content of xanthosine and xanthine in the skin of AD patients was 54 times and 38 times that of HV, and the content of L-phenylalanine and trans-cinnamate was approximately 4 times and 5 times that of HV, respectively, suggesting that the metabolic pathways related to xanthosine/xanthine and L-phenylalanine/trans-cinnamate are upregulated in AD. In addition, sphingosine levels were also significantly elevated in AD patients, approximately 9 times higher than in HV.

Similarly, KEGG pathway analysis of other subgroups was conducted (Supplementary Figures S5A–E). We found significant enrichment of differentially abundant metabolites in purine metabolism and phenylalanine metabolism pathways between ADF and HVF (Supplementary Figure S5A). The content of xanthosine and xanthine in ADF is 99 times and 75 times that of HVF, and the content of L-phenylalanine and trans-cinnamate is approximately 7 times and 9 times that of HVF, respectively. However, the comparison between ADM and HVM showed only differentially abundant metabolite enrichment in the purine metabolism pathway (Supplementary Figure S5B). The xanthosine and xanthine contents of ADM were 30 times and 7 times that of HVM, respectively. In addition, the differentially abundant metabolites of purine metabolism and phenylalanine metabolism pathways were also enriched between ADF and ADM (Supplementary Figure S5C). However, there was no significant difference in the above two pathways between HVF and HVM (Supplementary Figure S5D). Through the above subgroup analysis, it can be inferred that the skin metabolic phenotype of AD patients differed by sex, and the differences in the purine metabolism and phenylalanine metabolism pathways between AD patients and HV were more obvious in females.

### 3.4 Correlation analysis between the skin microbiota and skin metabolism

In total, 569 differentially abundant bacteria at the genus level and 81 differentially abundant metabolites at credibility levels of 1 to 3 between the AD group and HV group were obtained. Spearman correlation analysis was performed between the above differentially abundant bacteria and differentially abundant metabolites (Supplementary Table S6). Furthermore, the correlation between five important kinds of differentially abundant bacteria revealed by LEfSe analysis and the key differentially abundant metabolites in purine metabolism, phenylalanine metabolism and sphingolipid signaling pathways are shown in Table 2. We found that the genus *Staphylococcus* was positively correlated with various compounds in phenylalanine metabolism and purine metabolic pathways. The genera *Brevundimonas* and *Lactobacillus* were negatively correlated with various compounds in purine metabolism, phenylalanine metabolism and sphingolipid signaling pathways.

**Table 2:** The correlation between specific bacteria and differentially abundant metabolites.

Compound		*Staphylococcus*	*Brevundimonas*	*Propionibacterium*	*Lactobacillus*		*Lysobacter*
L-phenylalanine^a^		***0.610***	***−0.508***	−0.325	***−0.578***		−0.221
Trans-cinnamate^a^		***0.642***	***−0.541***	***−0.330***	***−0.578***		−0.266
Tran-2-hydroxycinnamate^a^		***0.570***	***−0.505***	−0.293	***−0.564***		−0.259
Phenylactate^a^		***0.591***	***−0.368***	−0.195	***−0.442***		−0.426
Xanthine^b^		***0.523***	***−0.436***	***−0.418***	−0.282		−0.096
Xanthosine^b^		***0.772***	***−0.549***	***−0.372***	***−0.467***		−0.244
Hypoxanthine^b^		−0.321	***0.421***	***0.416***	0.217		−0.033
Adenosine^b^		***0.448***	***−0.509***	0.027	***−0.354***		−0.278
Guanosine^b^		***0.430***	−0.295	−0.126	***−0.408***		***−0.338***
Deoxyinosine^b^		***0.437***	***−0.535***	−0.131	***−0.468***		−0.282
Sphingosine^c^		0.257	***−0.395***	−0.243	***−0.381***		−0.191
*r* ≤ −0.6	−0.6 < *r* ≤ −0.4	−0.4 < *r* ≤ −0.2	−0.2 < *r* ≤ 0	0 < *r* ≤ 0.2	0.2 < *r* ≤ 0.4	*0.4 < r ≤ 0.6*	*r*>0.6

Bold and italic values indicate nominal significant associations (*p* < 0.05).

a These metabolites are involved in phenylalanine metabolism.

b These metabolites are involved in purine metabolism.

c These metabolites are involved in sphingolipid signaling pathway.

## 4 Discussion

Skin microecological imbalance plays an important role in the pathogenesis of AD (Langan et al., 2020). The relationship between human and skin microbiota is bidirectional. Skin microbiota can affect host gene expression, and different skin characteristics can also form microbiota with specific preferences (Fyhrquist et al., 2019). There are also complex interactions between different skin bacteria. Previous studies have suggested that *S. aureus* is often dominant in the skin of AD patients, which can promote the onset of AD and limit the growth of other bacteria, which may have regulatory or protective effects (such as *Staphylococcus epidermidis* and *Corynebacterium*) (Fyhrquist et al., 2019). In contrast, some strains of *Staphylococcus epidermidis, Roseomonas* and *Propionibacterium* can inhibit the growth of *S. aureus* and are potential probiotics for the treatment of AD (Koh et al., 2022). Our study is based on 16S high-throughput sequencing technology, and the results are difficult to accurately annotate to the species level, nor can they be used to detect viruses or eukaryotic communities (Ghosh et al., 2018). Our study showed that the alpha diversity of the skin microbiota decreased in AD patients. The relative abundance of the genus *Staphylococcus* significantly increased, while the relative abundances of the genera *Propionibacterium*, *Brevundimonas*, *Lactobacillus* and *Lysobacter* significantly decreased, suggesting that the genus *Staphylococcus* affected the growth of other skin symbiotic flora and destroyed the skin microecological balance. Thus, it is reasonable to infer that *S. aureus* should account for most of the detected genus *Staphylococcus* and play an important role in the pathogenesis of AD.

In addition to disease factors, the skin bacterial structure is also affected by other confounding factors. It has been reported that the number of bacteria at the genus level does not change from birth to 1 year of age, but the relative abundance of microbiota fluctuates, with the relative abundances of the genera *Staphylococcus* and *Streptococcus* decreasing as infants grow, while the relative abundances of other genera increase (Capone et al., 2011). This trend was maintained through childhood (2–12 years old), with more diversity of skin microbiota on the forearms of children than adults (Shi et al., 2016). The composition of skin microbiota in adolescents was stable, similar to that of adults (Shi et al., 2016). The skin microbiome structure in different sites of AD was also different (Bjerre et al., 2021). Therefore, the subjects included in this study were all adults, and the sampling sites were all elbows, which can better reduce the interference of age and site factors on the research results. To investigate the effect of sex and local rash factors on the results, we performed a subgroup analysis. We revealed that there were significant differences in the composition and structure of the bacterial community between different sexes and lesion states.

Due to the important role of skin microbiota in the pathogenesis of AD, restoring skin microbial homeostasis is becoming a novel therapeutic strategy for treating AD (Hrestak et al., 2022; Koh et al., 2022). Application of prebiotics and probiotics, whether by gastrointestinal approach or external use, may help to increase the diversity of the skin microbiota and have potential therapeutic effect of AD (Hrestak et al., 2022). Coagulase-negative *Staphylococcus* (CoNS) strains with antimicrobial activity were common on the normal population but rare on AD subjects, and application of antimicrobial CoNS strains to human subjects with AD may decreased colonization by *S. aureus* (Nakatsuji et al., 2017). Topical microbiome transplantation with *Roseomonas mucosa* for AD patients was associated with significant decreases in measures of disease severity and *S. aureus* burden (Myles et al., 2018). A Living symbiotic bacteria-involved skin dressing was developed for microbiome-based biotherapy toward AD, which may recover skin barrier functions and alleviate AD-associated inflammation responses (Liu et al., 2023).

Samples for metabolomics analyses can be serum, urine, sweat, skin, etc. (Afghani et al., 2022). Both host gene expression products and substances produced by skin microorganisms influence skin metabolites (Afghani et al., 2022). Skin samples can be collected by invasive or noninvasive methods; the former is by skin biopsy, and the latter is by tape strips (Afghani et al., 2022). It has been reported that the swabbing method is comparable to the tape stripping method for collecting viable skin bacteria without losing fidelity to the composition of the skin microbiome (Ogai et al., 2018). However, to our knowledge, there have been no previous studies in AD metabolomics applying the swabbing method for sampling. In this study, a polyester swab was used to collect samples from skin, and large amounts of metabolites were successfully obtained. This noninvasive method for skin sample collection was proven to be safe, convenient and efficient.

Diet, age, sex, sampling method and other factors may affect the metabolites detected (Afghani et al., 2022). We evaluated the influence of disease, sex and rashes on the metabolomics analyses. The PCA results revealed that metabolites of healthy people have better consistency than those of AD patients, and males have better consistency than females. The number of differentially abundant metabolites *per capita* suggested that the effects of the state of AD, sex and the presence or absence of rashes on metabolomics analyses were successively reduced.

The differentially abundant metabolites between AD patients and HVs were enriched in the purine metabolism and phenylalanine metabolism pathways. The contents of xanthosine/xanthine and L-phenylalanine/trans-cinnamate are significantly elevated in AD, especially among females. However, we should notice that the metabolites we detected do not necessarily derived from microbes, they may also origin from the host. Xanthine is the substrate of xanthine oxidase and xanthine dehydrogenase, and it is also the intermediate product of the process from hypoxanthine to uric acid (Kulikowska et al., 2004). Xanthine is involved in a variety of intracellular metabolic pathways, such as purine nucleotide catabolism (Kulikowska et al., 2004). Exogenous guanosine and xanthosine, which are fluxed through the GTP branch of purine biosynthesis, were shown to significantly reduce methicillin-resistant *S. aureus* (MRSA) β-lactam resistance (Nolan et al., 2023). Further study demonstrated that exposure of MRSA to guanosine and xanthosine can significantly reduce the levels of the cyclic dinucleotide c-di-AMP, which is needed for β-lactam resistance (Nolan et al., 2023).

Phenylalanine is involved in a variety of metabolic activities in the body, and tyrosine, phenylactate and trans-cinnamate can be produced under the action of different enzymes (Matthews, 2007; Teufel et al., 2010; Otto et al., 2019). Bacteria in the environment can participate in the metabolism of phenylalanine and phenylacetate (Teufel et al., 2010). *Pseudomonas taiwanensis* has been used to catalyze trans-cinnamate formation from phenylalanine (Otto et al., 2019). The functional predictions based on metagenomic analysis in previous study revealed that AD skin samples exhibited enrichment in phenylalanine tyrosine and tryptophan biosynthesis (Chng et al., 2016). In addition, human lack the ability to synthesize essential amino acids such as phenylalanine, and they acquire essential amino acids from their diet or perhaps their associated microbial communities (Shrode et al., 2022; McCann and Rawls, 2023). Essential amino acid may act as chemical mediators of host–microbe interaction (McCann and Rawls, 2023). Thus, it was reasonable to infer that the significant proportion of increasing phenylalanine in AD we detected by skin metabolomics was probably derived from dominant microbes in AD. As far as we know, there are no relevant studies on purine metabolism and phenylalanine metabolism pathways in the pathogenesis of AD, but there is evidence that xanthosine, phenylalanine and trans-cinnamate participate in bacterial metabolic activities (Otto et al., 2019; Nolan et al., 2023). The important role of *S. aureus* in the pathogenesis of AD has been generally confirmed, and this study suggested that xanthosine/xanthine and L-phenylalanine/trans-cinnamate were positively correlated with *Staphylococcus*. Therefore, the purine metabolism and phenylalanine metabolism pathways may play a certain role in the pathogenic mechanism of *S. aureus* in AD, which is worthy of further exploration.

Epidermal barrier dysfunction is also a major characteristic of AD (Langan et al., 2020). Ceramide plays an important role in maintaining skin barrier function, and sphingosine is an important component of ceramide (Toncic et al., 2020). Ceramides can be divided into several subtypes, and they are dysregulated in AD patients (Ghosh et al., 2018). Previous studies have observed a decrease in long-chain ceramides and an increase in short-chain ceramides in AD patients (Afghani et al., 2022). It was reported that the colonization of *Staphylococcus* was positively correlated with the epidermal ceramide subspecies AS, ADS, NS and NDS in AD (Emmert et al., 2021). Our study showed that the amount of sphingosine in AD patients was approximately 10 times that in healthy controls (*p* < 0.05), which was consistent with previous studies (Toncic et al., 2020). However, we did not identify ceramide substances in this study, which may be due to the wide variety of ceramides and lack of a specialized ceramide comparison database.

There are some limitations in our study. First, the correlation between the skin microbiota and skin metabolites is only statistically significant, and it was difficult to determine whether the differential metabolites between the two groups detected in skin samples came from the host or microorganisms. Second, our study only detected the metabolites in skin samples without untargeted metabolomics analysis on serum samples, which can better reflect the metabolic situation of the body. It is necessary to collect both skin samples and blood samples in future research. In addition, relatively fewer subjects were included in the subgroup analysis, which inevitably increased the random error. Thus, the specific mechanism of interaction between skin microbiota, human skin and skin metabolites still needs to be further studied.

In summary, AD patients had dysbiosis of the skin microbiome with the features of decreased species richness and evenness. The relative abundance of the genus *Staphylococcus* increased significantly in AD, while the relative abundances of the genera *Propionibacterium*, *Brevundimonas*, *Lactobacillus* and *Lysobacter* were significantly decreased. Multiple metabolites related to purine metabolism and phenylalanine metabolism pathways (such as xanthosine/xanthine and L-phenylalanine/trans-cinnamate) were upregulated in AD patients, and they were positively correlated with the genus *Staphylococcus,* which suggested that purine metabolism and phenylalanine metabolism pathways may play a certain role in the pathogenic mechanism of *S. aureus* in AD. We also found that different sexes had certain effects on skin microbiota and metabolite composition. Females are more likely to be colonized by the genus *Staphylococcus* than males, and the differentially abundant metabolites involved in purine metabolism and phenylalanine metabolism pathways were more obvious in female.

## Data availability statement

The datasets presented in this study can be found in online repositories. The names of the repository/repositories and accession number(s) can be found in the article/Supplementary material.

## Ethics statement

The studies involving humans were approved by the Ethics Committee of Guangdong Provincial Hospital of Chinese Medicine (BE2019-165-01). The studies were conducted in accordance with the local legislation and institutional requirements. The participants provided their written informed consent to participate in this study.

## Author contributions

KH: Software, Writing – original draft, Visualization, Funding acquisition, Formal analysis. FL: Writing – original draft, Investigation, Data curation. YLiu: Writing – original draft, Investigation, Data curation. BL: Data curation, Writing – original draft, Investigation. PQ: Writing – original draft, Data curation. LY: Writing – original draft, Formal analysis. SH: Writing – original draft, Formal analysis. WL: Writing – review & editing, Project administration, Funding acquisition, Formal analysis. XM: Writing – original draft, Funding acquisition, Data curation. LD: Writing – review & editing, Supervision, Project administration, Funding acquisition, Conceptualization. YLin: Writing – review & editing, Supervision, Resources, Project administration, Funding acquisition, Conceptualization.
