# Supplementary material for: Multi-omics analyses reveal interactions between the skin microbiota and skin metabolites in atopic dermatitis
Source: Front Microbiol. 2024 Mar 15;15:1349674. doi: 10.3389/fmicb.2024.1349674 (PMC10978668; doi:10.3389/fmicb.2024.1349674)
Supplement: Supplementary file 2 [file Data_Sheet_1.PDF]

Supplementary Figures

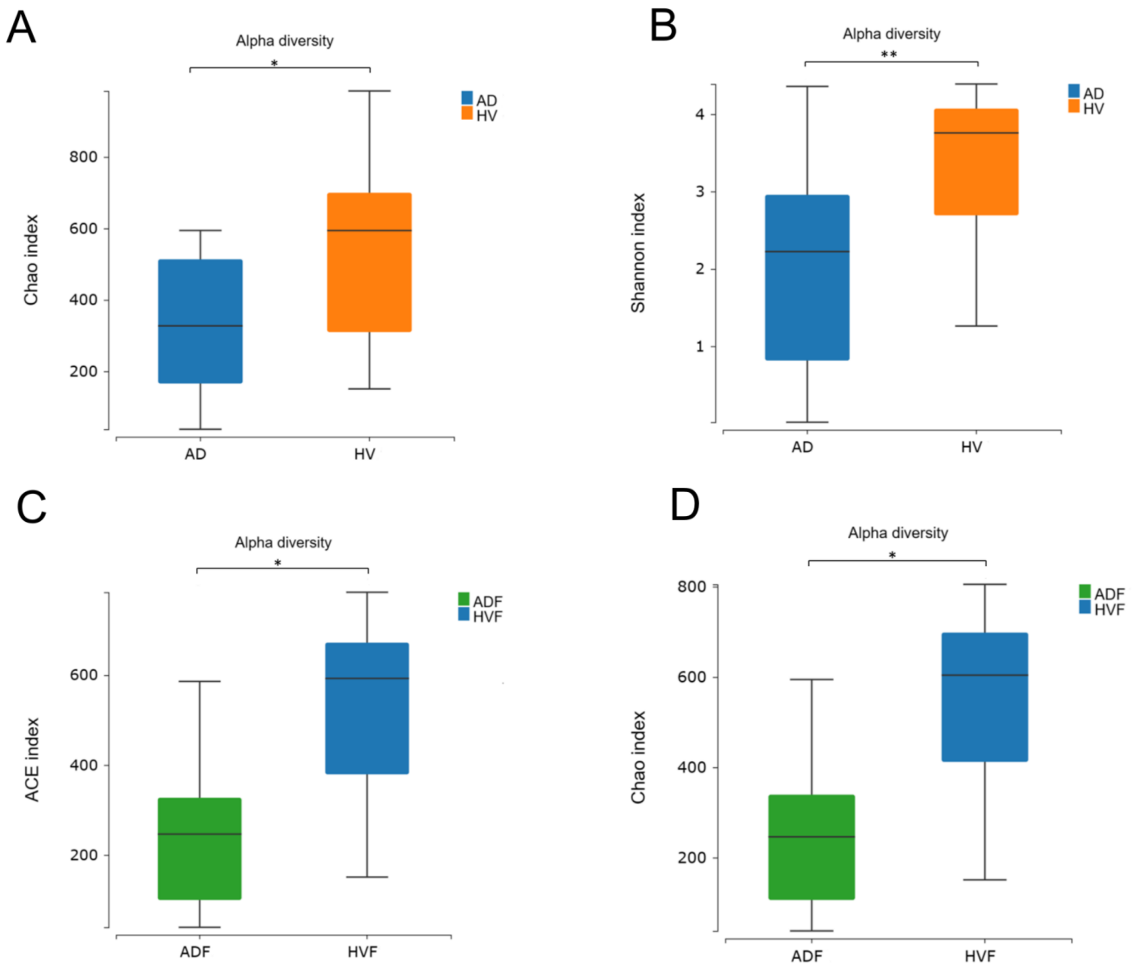

Supplementary Figure 1. Alpha diversity between some groups. (A) The Chao index between the AD group and HV group. (B) The Shannon index between the AD group and HV group. (C) The ACE index between the ADF group and HVF group. (D) The Chao index between the ADF group and HVF group. *$p < 0.05$; **$p < 0.01$.

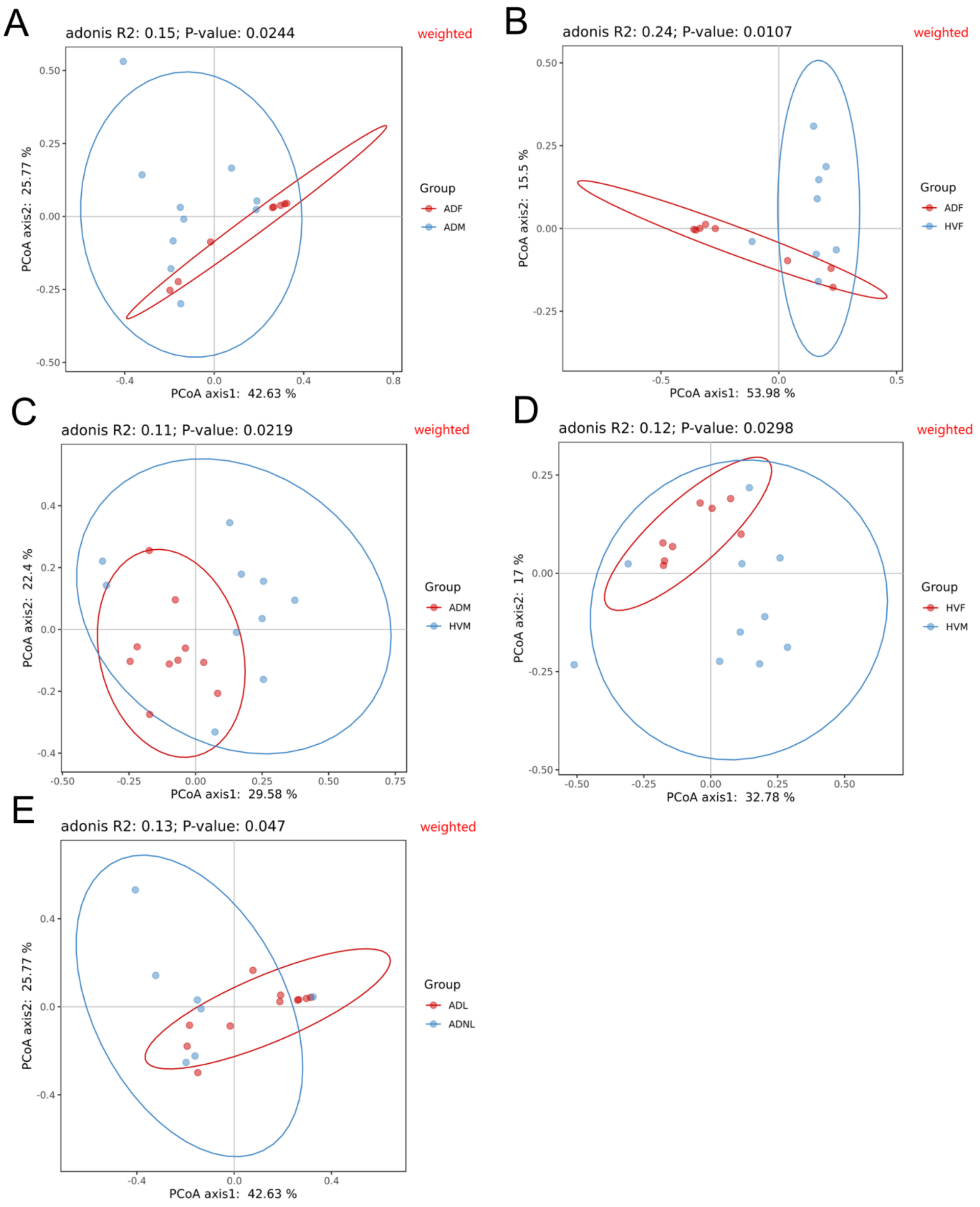

Supplementary Figure 2. Beta diversity analyzed by weighted Unifrac PCoA of all subgroups. (A) ADF group and ADM group. (B) ADF group and HVF group. (C) ADM group and HVM group. (D) HVF group and HVM group. (E) ADL group and ADNL group.

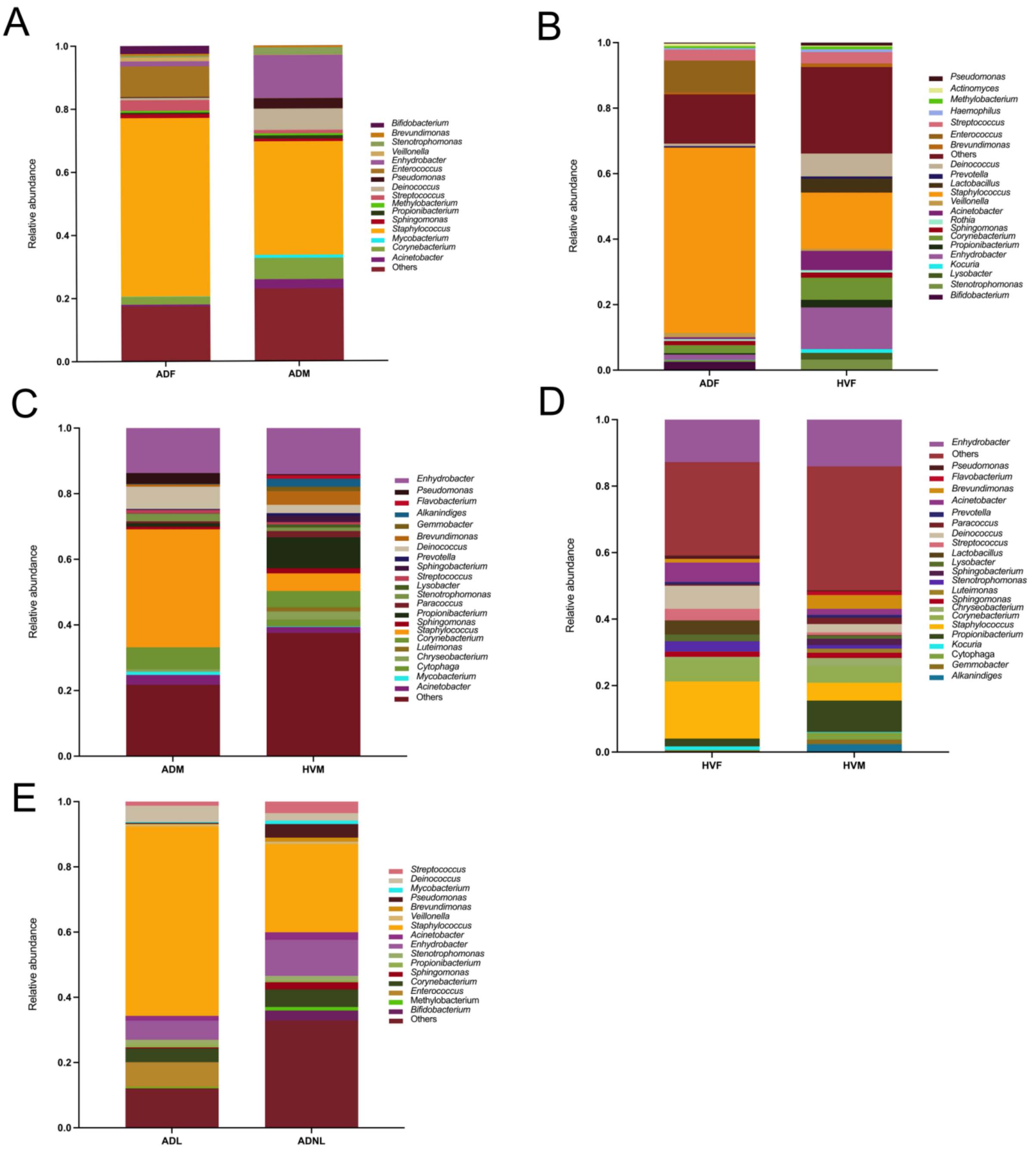

Supplementary Figure 3. Comparison of bacteria at the genus level in all subgroups. (A) ADF group and ADM group. (B) ADF group and HVF group. (C) ADM group and HVM group. (D) HVF group and HVM group. (E) ADL group and ADNL group.

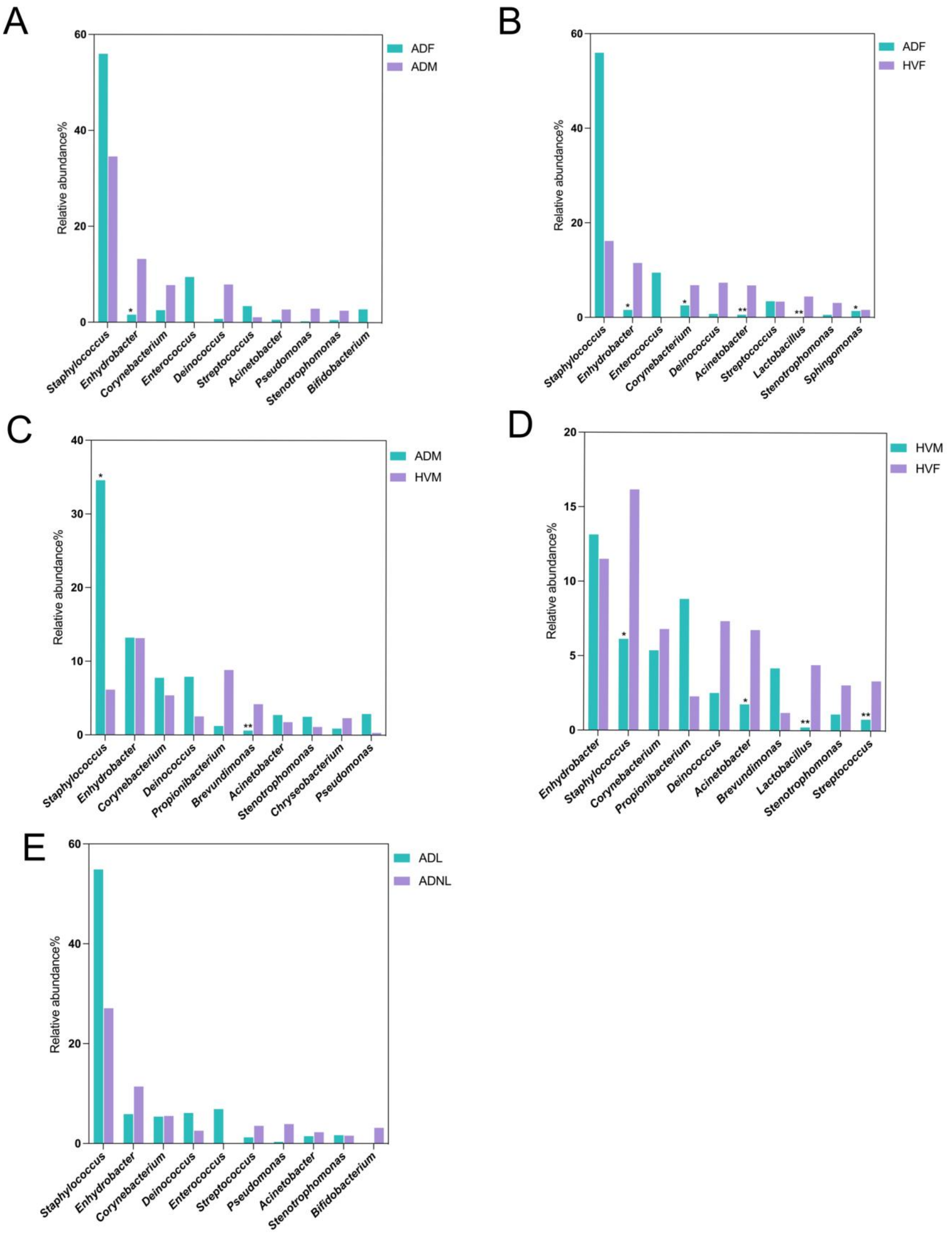

Supplementary Figure 4. Top 10 genera in terms of relative abundance in all subgroups. (A) ADF group and ADM group. (B) ADF group and HVF group. (C) ADM group and HVM group. (D) HVF group and HVM group. (E) ADL group and ADNL group. *$p < 0.05$; **$p < 0.01$.

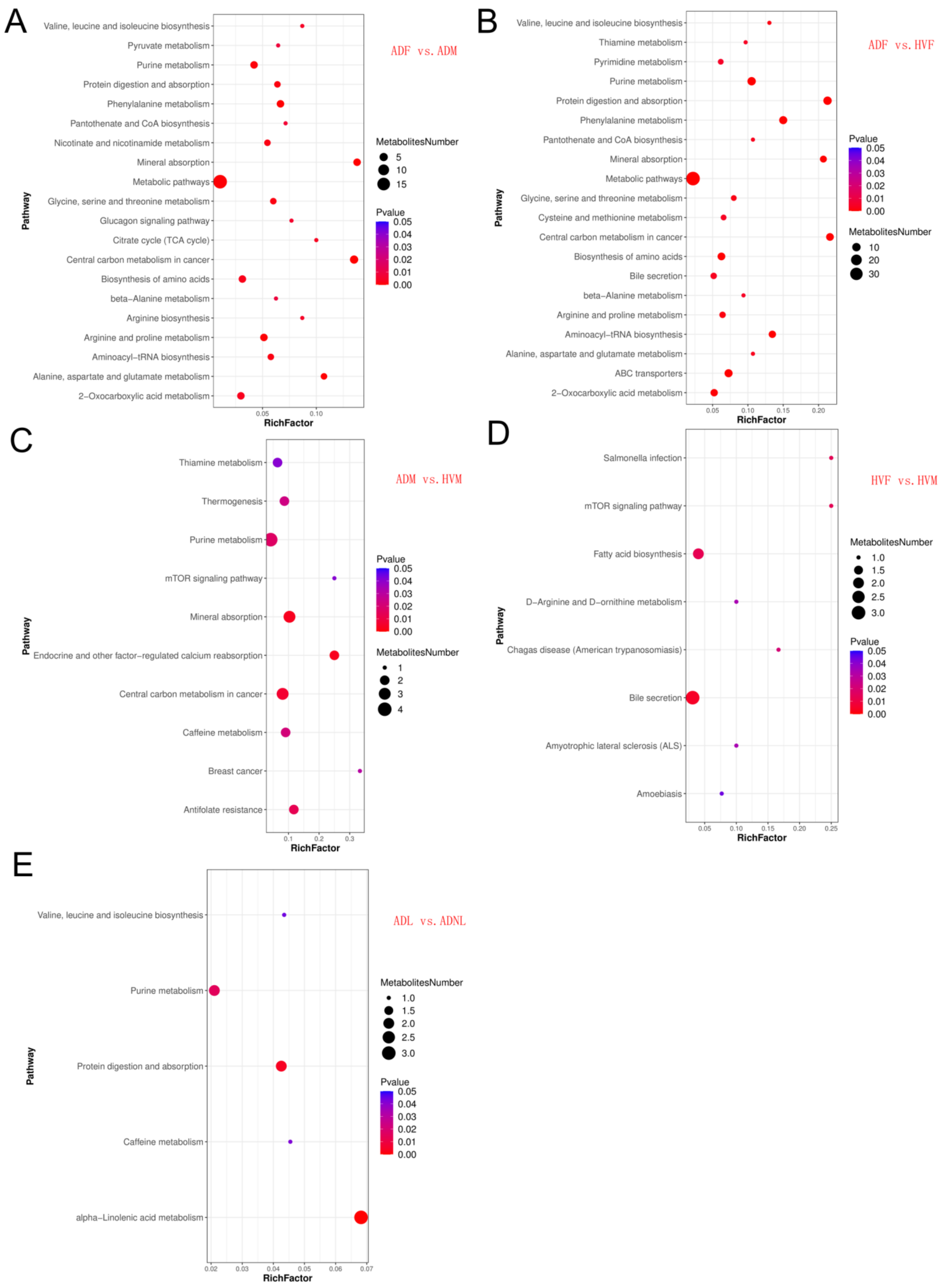
A
ADF vs. ADM
Valine, leucine and isoleucine biosynthesis
Pyruvate metabolism
Purine metabolism
Protein digestion and absorption
Phenylalanine metabolism
Pantothenate and CoA biosynthesis
Nicotinate and nicotinamide metabolism
Mineral absorption
Metabolic pathways
Glycine, serine and threonine metabolism
Glucagon signaling pathway
Citrate cycle (TCA cycle)
Central carbon metabolism in cancer
Biosynthesis of amino acids
beta−Alanine metabolism
Arginine biosynthesis
Arginine and proline metabolism
Aminoacyl−tRNA biosynthesis
Alanine, aspartate and glutamate metabolism
2−Oxocarboxylic acid metabolism
Pathway
RichFactor
0.05
0.10
MetabolitesNumber
5
10
15
Pvalue
0.05
0.04
0.03
0.02
0.01
0.00
B
ADF vs. HVF
Valine, leucine and isoleucine biosynthesis
Thiamine metabolism
Pyrimidine metabolism
Purine metabolism
Protein digestion and absorption
Phenylalanine metabolism
Pantothenate and CoA biosynthesis
Mineral absorption
Metabolic pathways
Glycine, serine and threonine metabolism
Cysteine and methionine metabolism
Central carbon metabolism in cancer
Biosynthesis of amino acids
Bile secretion
beta−Alanine metabolism
Arginine and proline metabolism
Aminoacyl−tRNA biosynthesis
Alanine, aspartate and glutamate metabolism
ABC transporters
2−Oxocarboxylic acid metabolism
Pathway
RichFactor
0.05
0.10
0.15
0.20
Pvalue
0.05
0.04
0.03
0.02
0.01
0.00
MetabolitesNumber
10
20
30
C
ADM vs. HVM
Thiamine metabolism
Thermogenesis
Purine metabolism
mTOR signaling pathway
Mineral absorption
Endocrine and other factor−regulated calcium reabsorption
Central carbon metabolism in cancer
Caffeine metabolism
Breast cancer
Antifolate resistance
Pathway
RichFactor
0.1
0.2
0.3
Pvalue
0.05
0.04
0.03
0.02
0.01
0.00
MetabolitesNumber
1
2
3
4
D
HVF vs. HVM
Salmonella infection
mTOR signaling pathway
Fatty acid biosynthesis
D−Arginine and D−ornithine metabolism
Chagas disease (American trypanosomiasis)
Bile secretion
Amyotrophic lateral sclerosis (ALS)
Amoebiasis
Pathway
RichFactor
0.05
0.10
0.15
0.20
0.25
MetabolitesNumber
1.0
1.5
2.0
2.5
3.0
Pvalue
0.05
0.04
0.03
0.02
0.01
0.00
E
ADL vs. ADNL
Valine, leucine and isoleucine biosynthesis
Purine metabolism
Protein digestion and absorption
Caffeine metabolism
alpha−Linolenic acid metabolism
Pathway
RichFactor
0.02
0.03
0.04
0.05
0.06
0.07
MetabolitesNumber
1.0
1.5
2.0
2.5
3.0
Pvalue
0.05
0.04
0.03
0.02
0.01
0.00

Supplementary Figure 5. Bubble plot of the metabolic pathway enrichment analysis results of all subgroups. (A) ADF group and ADM group. (B) ADF group and HVF group. (C) ADM group and HVM group. (D) HVF group and HVM group. (E) ADL group and ADNL group.
